# Optimization of Sweet Leaf (*Sauropus androgynus* L. Merr)–Amaranth (*Amaranthus hybridus* L.) Vegetable Leather With Carrageenan and Sorbitol

**DOI:** 10.1155/sci5/5572976

**Published:** 2026-01-08

**Authors:** Heri Purwoto, Anjani Putri Purnamasari, Tubagus Bahtiar Rusbana, Winda Nurtiana, Renny Primasari Gustia Putri, Maya Soraya, Hendrawan Laksono

**Affiliations:** ^1^ Research Center for Process Technology, National Research and Innovation Agency (BRIN), KST BJ Habibie Setu, South Tangerang, Banten, 15314, Indonesia, brin.go.id; ^2^ Department of Food Technology, Faculty of Agriculture, University of Sultan Ageng Tirtayasa, Serang, 42110, Indonesia

**Keywords:** amaranth leaf, carrageenan, sorbitol, sweet leaf, vegetable leather

## Abstract

Vegetable leather is an emerging preservation strategy that extends shelf life, reduces postharvest losses, and provides a convenient format for nutrient‐rich vegetables. Sweet leaf (*Sauropus androgynus* L. Merr.) and amaranth (*Amaranthus hybridus* L.) are rich in bioactive compounds, making them promising raw materials for functional vegetable leather. This study optimized vegetable leather formulation by evaluating the effects of carrageenan, sorbitol, and sweet leaf–amaranth ratios on mechanical and physicochemical properties. Response surface methodology (RSM) with a D‐optimal design was applied using Design Expert 13.0.12 software. Factors tested included carrageenan concentration (0.5%–2.5%), sorbitol concentration (1%–13%), and sweet leaf–amaranth ratios (25:75, 50:50, and 75:25). Optimization results identified the optimal formula as 0.877% carrageenan, 1% sorbitol, and a 25:75 sweet leaf–amaranth ratio, with a desirability value of 0.864. The optimized product exhibited tensile strength of 6.08 N/mm^2^, elongation of 6%, solubility of 92.77%, moisture content of 10.93%, and ash content of 3.11%. Functional analysis of the optimized product showed a chlorophyll content of 9.35 ± 0.35 mg·L^−1^ and antioxidant activity of 76.0 ± 0.02% inhibition. Sensory evaluation indicated neutral to slightly favorable acceptance, with overall acceptability of 5.1 ± 0.90 on a seven‐point hedonic scale. These results confirm that the optimized sweet leaf–amaranth vegetable leather has promising functional and consumer attributes in addition to desirable physicochemical properties.

## 1. Introduction

Fruits and vegetables undergo physicochemical changes after harvest, leading to nutrient degradation, moisture loss, and increased susceptibility to microbial contamination. These postharvest changes are primarily influenced by environmental factors, which accelerate spoilage and contribute to significant food losses. Microbial contamination, particularly from fungi such as *Aspergillus niger*, *Rhizopus oryzae*, and *Botrytis cinerea*, further reduces shelf life and affects food safety.

To mitigate these losses, vegetable leather has emerged as an emerging preservation method. This product is created by dehydrating a mixture of vegetable pulp and additives into a flexible, sheet‐like form, offering an extended shelf life while retaining essential nutrients. Vegetable leather has been developed using various vegetable sources, including amaranth, broccoli, and carrots, demonstrating its potential as a functional and convenient food product. However, achieving the desired texture, flexibility, and mechanical properties in vegetable leather depends on the selection and optimization of binding agents and plasticizers.

Carrageenan, a galactose polysaccharide extracted from *Eucheuma* sp., is widely used as a gelling agent and texture stabilizer in food applications. It enhances gel strength, elasticity, and water retention, contributing to a flexible and cohesive product structure [1, 2]. In contrast, sorbitol or sugar alcohol (polyol), a commonly used plasticizer, plays a crucial role in reducing intermolecular hydrogen bonding, improving flexibility, and preventing brittleness in dried food products [3]. However, excessive sorbitol addition can lead to an overly soft and sticky texture, whereas insufficient sorbitol results in a rigid and brittle product [4–6].

Despite previous studies on vegetable leather, a systematic approach to optimizing its formulation remains limited. The balance between binding agents and plasticizers must be carefully controlled to achieve optimal texture and mechanical performance. Therefore, this study aims to optimize the formulation of vegetable leather by evaluating the effects of carrageenan, sorbitol, and different sweet leaf‐to‐amaranth ratios. The response surface methodology (RSM) using Design Expert 13.0.12 software will be employed to determine the optimal composition that ensures a flexible, stable, and high‐quality product.

## 2. Materials and Methods

### 2.1. Raw Materials

Young sweet leaves (*Sauropus androgynus* L. Merr.) and amaranth leaves (*Amaranthus hybridus* L.) were purchased from Lembang Market, Tangerang, Banten, Indonesia. Young leaves were selected because they contain higher levels of chlorophyll and phenolic compounds, have softer textures, and yield smoother purees, which are advantageous for uniform film formation in vegetable leather. Kappa carrageenan (food‐grade, Indoflora Ltd., Indonesia) was used as the gelling agent, and 70% sorbitol (Bratachem, Indonesia) served as the plasticizer. Analytical‐grade reagents, including 2,2‐diphenyl‐1‐picrylhydrazyl (DPPH; Sigma Aldrich, USA), acetone (85%), and methanol (Supelco, Germany), were used for chlorophyll and antioxidant analyses. Additional ingredients included distilled water, sugar, salt, and seasoning.

### 2.2. Experimental Design

This study utilized an experimental design to optimize the formulation of vegetable leather by varying carrageenan concentration, sorbitol concentration, and the ratio of sweet leaf to amaranth. The analysis employed factor‐response evaluation using RSM in Design Expert 13.0.12 software. The experimental treatments included three variables: carrageenan concentration, sorbitol concentration, and the ratio of sweet leaf to amaranth, as detailed in Table 1.

**Table 1:** Testing levels for each variable.

Variable	Levels				
Carrageenan (%)	0.5	1.0	1.5	2.0	2.5
Sorbitol (%)	1	4	7	10	13
Sweet leaf:amaranth (%)	75:25		50:50		25:75

Regression analysis was performed using polynomial equations obtained from the RSM–D’Optimal method in Design Expert software. Experimental conditions were randomized, and each formula was tested in triplicate, resulting in 20 experimental units (Table 2). The optimization focused on tensile strength, elongation, solubility, ash content, and moisture content.

**Table 2:** RSM‐based vegetable leather formulation design.

Run	Carrageenan (%)	Sorbitol (%)	Sweet leaf:amaranth (%)
1	1.5	1	25:75
2	2.5	1	25:75
3	2.0	10	25:75
4	1.5	4	25:75
5	2.0	4	75:25
6	1.5	13	50:50
7	0.5	13	25:75
8	1.0	10	50:50
9	1.0	10	75:25
10	2.5	13	75:25
11	0.5	7	50:50
12	2.0	4	75:25
13	0.5	1	75:25
14	1.0	4	25:75
15	0.5	1	50:50
16	0.5	7	75:25
17	1.0	4	25:75
18	2.5	7	50:50
19	2.5	13	50:50
20	1.5	1	50:50

The experimental design allowed for the identification of the optimal formulation based on a comprehensive evaluation of mechanical, chemical, and sensory properties.

### 2.3. Vegetable Leather Preparation

The production process began with sorting and cleaning of sweet leaf and amaranth to remove stems and damaged parts, retaining only the leaf blades. The main ingredients were weighed according to the experimental design shown in Table 3. The leaves were steam‐blanched at 60°C for 5 min to inactivate enzymes and preserve color. Carrageenan and sorbitol were dissolved in 100 mL of distilled water, based on a total water volume of 500 mL. The blanched leaves were blended with the carrageenan–sorbitol solution, 4 g salt, 3 g seasoning, 15 g sugar, and the remaining 400 mL of water for 1 min until a homogeneous puree was obtained.

**Table 3:** Formulation of vegetable leather composing materials for 500 g.

Run	The ingredient (gram)							
	Sweet leaves	Amaranth leaves	Carrageenan	Sorbitol	Water	Salt	Flavor enhancer	Sugar
1	12.5	37.5	7.5	5	415.5	4	3	15
2	12.5	37.5	12.5	5	410.5	4	3	15
3	12.5	37.5	10	50	368	4	3	15
4	12.5	37.5	7.5	20	400.5	4	3	15
5	37.5	12.5	10	20	398	4	3	15
6	25	25	7.5	65	355.5	4	3	15
7	12.5	37.5	2.5	65	360.5	4	3	15
8	25	25	5	50	373	4	3	15
9	37.5	12.5	5	50	373	4	3	15
10	37.5	12.5	12.5	65	350.5	4	3	15
11	25	25	2.5	35	390.5	4	3	15
12	37.5	12.5	2	20	406	4	3	15
13	37.5	12.5	2.5	5	420.5	4	3	15
14	12.5	37.5	5	20	403	4	3	15
15	25	25	2.5	5	420.5	4	3	15
16	37.5	12.5	2.5	35	390.5	4	3	15
17	12.5	37.5	5	20	403	4	3	15
18	25	25	12.5	35	380.5	4	3	15
19	25	25	12.5	65	350.5	4	3	15
20	25	25	7.5	5	415.5	4	3	15

The puree was heated in a water bath at 80°C while stirring at 200 rpm to initiate partial gelation and reduce excess moisture. Subsequently, 200 mL of the mixture was poured into flat steel molds (34.4 × 24.4 × 2.3 cm) to achieve a target thickness of 0.1–0.2 mm. Drying was performed in a cabinet dryer at 50°C for 21 h until a flexible, nonsticky sheet was formed. The resulting sheets were cooled to room temperature, peeled from the mold, and stored in airtight containers for further analysis.

This formulation ensures consistent physical, chemical, and sensory characteristics in vegetable leather production.

### 2.4. Analyses

#### 2.4.1. Tensile Strength and Elongation

Tensile strength measures the force required to break vegetable leather. Samples are cut according to ASTM standards and tested at 27°C using a universal testing machine (UTM). The machine applies tension at a speed of 20 mm/min until rupture. Tensile strength (MPa) is calculated using the following equation:

(1) Tensile strength=FmaxA,

where *F*_max_ = maximum force applied (N) and *A* = cross‐sectional area (mm^2^).

Elongation represents the percentage increase in length before rupture. Samples are tested at 27°C using a UTM. The initial length is measured before applying tension, and the final length is recorded after rupture [7]. The percentage elongation is calculated as follows:

(2) %Elongation=P2−P1P1×100%,

where *P*_1_ = initial length (mm) and *P*_2_ = final length after rupture (mm).

#### 2.4.2. Solubility, Moisture, and Ash Content

Solubility indicates the ease with which vegetable leather dissolves in water. Samples are cut into 2 × 2 cm pieces and dried at 105°C for 24 h. The initial weight (*W*_1_) is recorded. Each sample is then placed in a centrifuge tube with 10 mL of distilled water and shaken at 27°C for 24 h. The mixture is filtered, and the residue is dried at 105°C for another 24 h [8]. The final weight (*W*_2_) is recorded, and solubility is calculated using the following equation:

(3) % Solubility=W2−W1W1×100%,

where *W*_1_ = initial weight (g) and *W*_2_ = final weight (g).

Moisture content is determined gravimetrically. Samples are weighed, dried at 110°C until constant weight, and reweighed [9]. The moisture content is calculated using the following equation:

(4) %Moisture content=W1−W2W1−W0×100%,

where *W*_0_ = weight of empty dish (g), *W*_1_ = weight of dish with sample before drying (g), and *W*_2_ = weight of dish with sample after drying (g).

Ash content is measured using the dry ashing method. Samples are heated at 600°C until only inorganic residue remains [9]. The ash content is calculated using the following equation:

(5) %Ash content=Ash weightSample weight×100%.

### 2.5. Analysis of Optimal Vegetable Leather Formula

The response parameters for the best formulations include chlorophyll content testing, antioxidant activity testing, and organoleptic testing.

#### 2.5.1. Chlorophyll Content

Chlorophyll content was determined according to the method of Arnon [10] with minor modifications. The sample (5 g) was cut into small pieces and macerated in 100 mL of 85% acetone for 72 h (3 × 24 h) at room temperature. The mixture was filtered through Whatman No. 40 filter paper, and the filtrate was collected. Absorbance was measured at 645 and 663 nm using a UV–Vis spectrophotometer calibrated with 85% acetone as the blank. Chlorophyll a, chlorophyll b, and total chlorophyll were calculated using the standard equations:

(6) Chl a=12.72.69A633−A645,Chl b=22.94.68A645−A633,Total Chl=Chl a+Chl b.

Values were expressed as mg·L^−1^.

#### 2.5.2. Antioxidant Activity

Antioxidant activity was determined using the DPPH radical‐scavenging assay following the method of Brand‐Williams et al. [11] with modifications. A 0.1 mM DPPH solution was prepared by dissolving 0.0039 g DPPH in 100 mL of methanol and stored in a dark bottle wrapped with aluminum foil. A 1000 ppm stock sample solution was prepared by dissolving 100 mg of the sample in 100 mL of methanol, followed by serial dilutions (0–800 ppm). Each sample (2 mL) was mixed with 2 mL of 0.1 mM DPPH, vortexed, and incubated in the dark for 30 min. Absorbance was read at 517 nm using a UV–Vis spectrophotometer. Antioxidant activity (% inhibition) was calculated as follows:

(7) % Inhibition=A0−A0−A1A0×100,

where *A*_0_ = absorbance of the blank and *A*_1_ = absorbance of the sample.

#### 2.5.3. Organoleptic Evaluation

Sensory evaluation was carried out using a 7‐point hedonic scale with 35 untrained panelists (men and women, aged 18–50 years). Panelists evaluated the aroma, taste, color, texture, and overall acceptability of the optimized vegetable leather. The preference scale ranged from 1 = very dislike to 7 = very like. Mean scores and standard deviations were calculated for each attribute.

### 2.6. Data Processing

#### 2.6.1. Response Analysis Stage

Data analysis is performed based on the average measurement values of the 20 experimental units following the designated design. The analysis of variance (ANOVA) results are evaluated to determine the significance of the model, ensuring that the model significance is confirmed while the lack of fit remains nonsignificant. This verification helps assess the meaningful effect of the treatments on the response. Additionally, reasonable agreement between the adjusted *R*‐squared and predicted *R*‐squared values is maintained when their difference is less than 0.2. Furthermore, adequate precision should exceed a value of 4 to indicate a desirable signal‐to‐noise ratio, ensuring the reliability of the statistical model.

#### 2.6.2. Optimization Stage

After analyzing the data from the 20 experimental units, the response of each analysis is used to determine the optimal formula using Design Expert 13.0.12 software. The optimization process considers parameters, such as tensile strength, elongation, solubility, moisture content, and ash. The software recommends several optimal formulations based on the maximum desirability value, ideally approaching 1. A desirability value close to 1 indicates that the response surface method can effectively optimize the formula with a high level of confidence. However, the objective is not only to achieve a desirability value of precisely one but also to identify the best conditions that satisfy all objective functions [12–14].

## 3. Results and Discussion

The production of vegetable leather using sweet leaves, amaranth leaves, carrageenan, and sorbitol follows the formulation recommended by the RSM–D’Optimal design expert program. The carrageenan substitution ranges from 0.5% to 2.5%, based on Sabila et al. [2], who identified 1.5% carrageenan as optimal for physical and chemical properties. Anggraini [15] also found that carrageenan acts as a gel strength stabilizer, with a narrow effective range (0.4%, 0.6%, and 0.8%), producing no significant differences in fruit leather’s physical properties. Therefore, this study employs a broader range to assess significant differences in response to each parameter.

Sorbitol addition ranges from 1% to 13%, with a threshold interval of 3%. This range is determined based on Haryu et al. [16] findings, which showed that 9.8% sorbitol yielded optimal fruit leather characteristics. As a humectant, sorbitol binds water, stabilizing moisture content and affecting texture. The interaction between sorbitol and water occurs through hydrogen bonding of O and H groups, leading to a softer and less rigid texture as sorbitol concentration increases [4, 17]. To prevent excessive softening and fragility, the upper limit is set at 13%.

The proportion of sweet leaves to amaranth leaves ranges from 25%:75%–75%:25%. These thresholds are based on Ramadhani and Saidi’s [18] study on mixed fruit and vegetable leather with carrageenan. Their research explored ratios of 25%:75%, 50%:50%, and 75%:25% for broccoli and jackfruit, finding the 25%:75% ratio optimal for physical and chemical properties. Given the limited research on vegetable leather using two vegetable types, this study aims to introduce a combination for vegetable leather production.

The analyzed parameters include tensile strength, elongation, solubility, color intensity (chroma and HUE), moisture content, ash content, and water activity. The response analysis results for the 20 formulations are presented in Table 4.

**Table 4:** Results of response analysis of vegetable leather formulations using Design Expert 13.02 software.

Run	Kg^1^	Sb^2^	K:S^3^	TS^4^	Elo^5^	Solu^6^	Ash	MC^7^
	%	%	%	(N/mm^2^)	(%)	(%)	(%)	(%)
13	0.5	1	75:25	3.04	2.29	98.52	2.48	11.53^∗^
15	0.5	1	50:50	3.56	3.54	98.39	2.18	10.13^∗^
16	0.5	7	75:25	4.34	4.52	95.34	2.57	10.36^∗^
11	0.5	7	50:50	4.92	5.42	92.26	2.70	11.09^∗^
7	0.5	13	25:75	5.84	6.75	87.40	2.91	12.27^∗^
14	1	4	25:75	6.40	7.73	85.85	3.42	11.21^∗^
17	1	4	25:75	6.88	7.60	86.33	3.09	11.09^∗^
9	1	10	75:25	7.05	8.98	85.20	2.18	12.98^∗^
8	1	10	50:50	7.38	10.61	87.51	3.04	11.76^∗^
20	1.5	1	50:50	15.38	17.80	62.37	8.87	10.56^∗^
1	1.5	1	25:75	7.27	9.60	79.76	3.06	10.34^∗^
4	1.5	4	25:75	7.67	10.29	76.12	3.49	10.59^∗^
6	1.5	13	50:50	7.87	12.18	71.38	4.00	10.36^∗^
12	2	4	75:25	8.05	10.15	70.62	4.52	10.91^∗^
3	2	10	25:75	8.73	11.37	67.87	5.05	11.95^∗^
5	2	4	75:25	9.64	12.41	67.38	5.25	10.30^∗^
2	2.5	1	25:75	12.01	10.92	65.91	6.83	10.11^∗^
18	2.5	7	50:50	12.04	13.81	63.62	5.53	11.26^∗^
10	2.5	13	75:25	15.55	15.43	62.34	8.27	10.67^∗^
19	2.5	13	50:50	7.34	8.18	80.20	5.04	10.46^∗^

^1^Carrageenan.

^2^Sorbitol.

^3^Sweet:amaranth.

^4^Tensile strength.

^5^Elongation.

^6^Solubility.

^7^Moisture content.

^∗^Nonsignificant influence.

### 3.1. Tensile Strength

Tensile strength is a fundamental mechanical property that helps in characterizing the behavior of materials under stress. It is crucial for selecting materials for engineering applications and developing new materials or processes [19]. Based on the results of the ANOVA on the tensile strength response data using the Design Expert 13.0.2 program, Table 5 shows that the linear model is the suggested model.

**Table 5:** Model summary statistics of tensile strength response.

Source	Sequential *p*‐value	Lack of fit *p*‐value	Adjusted *R* ^2^	Predicted *R* ^2^	
Linear	0.0096	0.0892	0.4538	0.1285	Suggested
2FI	0.0959	0.1241	0.6412	−0.2865	
Quadratic	0.4723	0.1133	0.6282	−2.9725	
Cubic	0.1133		0.9416		Aliased

**Figure 1:**
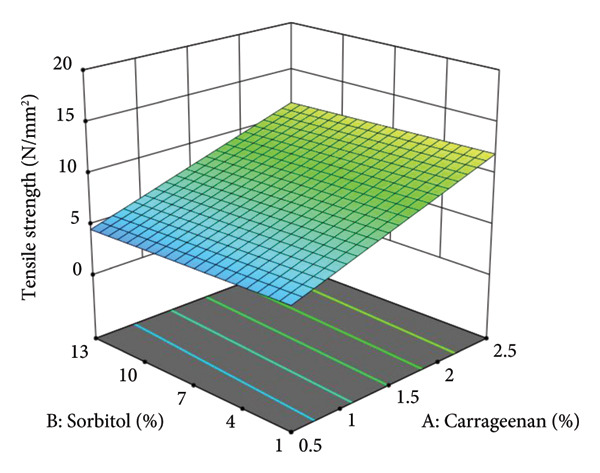
Three‐dimensional surface response curve of tensile strength.

The statistical analysis confirmed that the composition of vegetable leather significantly influences its mechanical properties, specifically tensile strength. This is indicated by a significant model and an insignificant lack of fit (Table 6), demonstrating the reliability of the regression model in predicting tensile strength. The interaction between carrageenan, sorbitol, and the sweet leaf–amaranth ratio plays a key role in determining the final product’s structural integrity. The variation in tensile strength, ranging from 3.04 to 15.55 N/mm^2^, highlights the importance of optimizing the formulation to achieve desirable mechanical characteristics. Additionally, the three‐dimensional response surface plot generated using Design Expert provides a comprehensive visualization of these interactions, illustrating how each factor contributes to the tensile strength of vegetable leather, as shown in Figure 1.

**Table 6:** ANOVA linear model for tensile strength response.

Source	Sum of squares	df	Mean square	*F*‐value	*p*‐value	
Model	128.02	4	32.00	4.95	0.0096	Significant
A‐Carrageenan	126.05	1	126.05	19.48	0.0005	
B‐Sorbitol	0.5993	1	0.5993	0.0926	0.7650	
C‐Sweet:amaranth	1.31	2	0.6537	0.1010	0.9045	
Residual	97.05	15	6.47			
Lack of fit	95.67	13	7.36	10.63	0.0892	Not significant
Pure error	1.38	2	0.6922			
Cor total	225.07	19				

The three‐dimensional graph shows a linear increasing trend in tensile strength with higher carrageenan concentrations. The tensile strength observed in this study may be attributed to the use of carrageenan concentrations up to 2.5%, which exceeds the levels used in previous studies. Carrageenan strengthens the molecular bonds within sweet and amaranth leaf vegetable leather, resulting in a more cohesive matrix [20]. A primary component of carrageenan facilitates gel formation and enhances gel strength, ultimately influencing the tensile strength of vegetable leather [20–22].

Figure 1 illustrates the tensile strength values based on carrageenan and sorbitol addition, averaged across sweet leaf–amaranth ratios of 75:25, 50:50, and 25:75. The graph shows a linear decrease in tensile strength with increasing sorbitol concentration, indicating that higher sorbitol levels reduce the material’s structural integrity. This trend aligns with the findings of Afdal et al. [23] and Rosmawati et al. [24], who reported that sorbitol functions as a plasticizer, weakening molecular interactions and thereby decreasing tensile strength.

### 3.2. Elongation to Break

The purpose of measuring elongation to break for biomaterials is to assess their mechanical properties, particularly ductility and toughness. This measurement helps in understanding how a biomaterial behaves under stress [25]. Based on the results of the ANOVA on the tensile strength response data using the Design Expert 13.0.2 program, Table 7 shows that the linear model and quadratic model are the suggested models.

**Table 7:** Model summary statistics of elongation to break response.

Source	Sequential *p*‐value	Lack of fit *p*‐value	Adjusted *R* ^2^	Predicted *R* ^2^	
Linear	0.0207	0.1133	0.3884	0.0554	Suggested
2FI	0.2956	0.1198	0.4644	−0.8740	
Quadratic	0.0244	0.2199	0.7353	−1.2928	Suggested
Cubic	0.2199		0.9159		Aliased

**Figure 2:**
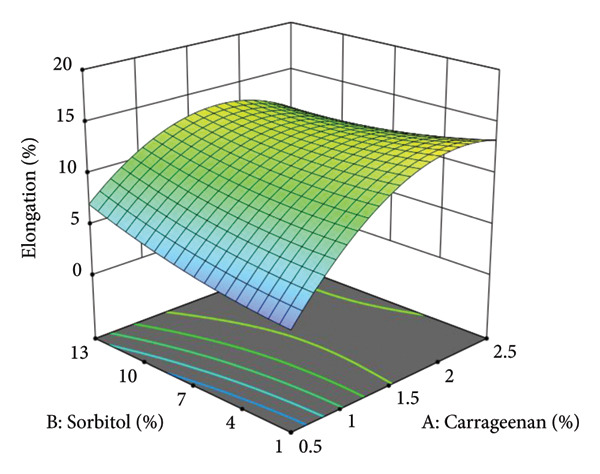
Three‐dimensional surface response curve of elongation.

The mechanical properties of vegetable leather, particularly elongation to break, are significantly influenced by its composition. This is evidenced by a statistically significant model with an insignificant lack of fit (Table 8), confirming the accuracy of the regression model in predicting elongation behavior. The interaction between carrageenan, sorbitol, and the sweet leaf–amaranth ratio plays a crucial role in determining the flexibility and structural integrity of the final product. Elongation values ranged from 2.29% to 17.80%, emphasizing the need for precise formulation adjustments to achieve optimal mechanical performance. Furthermore, the three‐dimensional response surface plot generated using Design Expert visually represents these interactions, illustrating how formulation factors affect elongation to break, as depicted in Figure 2.

**Table 8:** ANOVA quadratic model for elongation to break response.

Source	Sum of squares	df	Mean square	*F*‐value	*p*‐value	
Model	256.31	11	23.30	5.80	0.0097	Significant
A‐Carrageenan	128.67	1	128.67	32.02	0.0005	
B‐Sorbitol	0.3119	1	0.3119	0.0776	0.7876	
C‐Sweet:amaranth	14.79	2	7.40	1.84	0.2200	
AB	13.28	1	13.28	3.30	0.1067	
AC	2.40	2	1.20	0.2991	0.7494	
BC	35.60	2	17.80	4.43	0.0507	
*A* ^2^	49.13	1	49.13	12.23	0.0081	
*B* ^2^	1.62	1	1.62	0.4031	0.5432	
Residual	32.15	8	4.02			
Lack of fit	29.60	6	4.93	3.86	0.2199	Not significant
Pure error	2.55	2	1.28			
Cor total	288.46	19				

The graph indicates that elongation to break peaks at approximately 2% carrageenan concentration, followed by a decline with further carrageenan addition. Avila et al. [26] also reported that higher carrageenan levels result in a stronger film matrix, reducing elasticity and making the vegetable leather more brittle, thereby lowering elongation percentages.

The graph further illustrates a decline in elongation values with increasing sorbitol concentration, as indicated by the blue regions. However, Figure 2 also highlights an increase in elongation with higher sorbitol concentrations, suggesting that sorbitol enhances flexibility. This improvement is attributed to a reduction in activation energy required for molecular movement within the matrix, which enhances elasticity. Plasticizers, such as sorbitol, lower activation energy for molecular motion, thereby increasing flexibility. As a result, increasing sorbitol concentration up to a certain point improves elongation properties [27, 28].

### 3.3. Solubility

The solubility of vegetable leather in water is an important characteristic that influences its chewability, storage stability, and overall quality. Solubility determines how the product interacts with moisture during consumption and storage, thereby affecting texture and shelf life. This property is affected by factors such as the chemical composition and the interaction between carrageenan, sorbitol, and the vegetable matrix [29]. ANOVA using Design Expert 13.0.2 indicated that both linear and quadratic models were significant for predicting solubility behavior. The results are presented in Table 9. The model adequately described the relationship between ingredient levels and solubility, allowing prediction of how carrageenan and sorbitol concentrations influence the water‐binding capacity of the vegetable leather.

**Table 9:** Model summary statistics of solubility response.

Source	Sequential *p*‐value	Lack of fit *p*‐value	Adjusted *R* ^2^	Predicted *R* ^2^	
Linear	0.0002	0.0368	0.6800	0.5028	Suggested
2FI	0.2177	0.0421	0.7419	0.1720	
Quadratic	0.0842	0.0584	0.8262	−0.6195	Suggested
Cubic	0.0584		0.9862		Aliased

**Figure 3:**
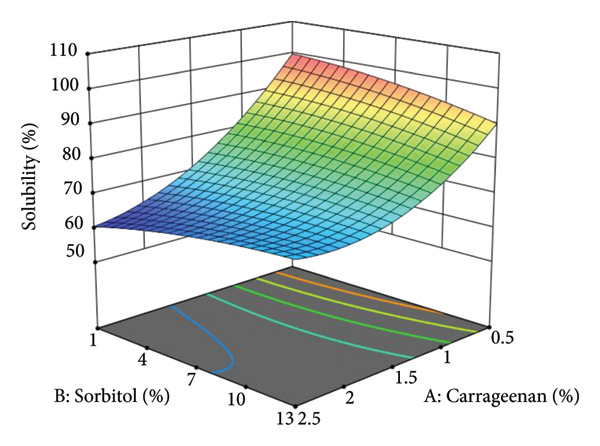
Three‐dimensional surface response curve of solubility.

The solubility of vegetable leather is highly dependent on its composition. This is supported by a statistically significant model with an insignificant lack of fit (Table 10), validating the regression model’s accuracy in predicting solubility behavior. The interaction among carrageenan, sorbitol, and the sweet leaf–amaranth ratio plays a vital role in shaping the structural integrity and flexibility of the final product. Solubility values varied between 2.05% and 98.52%, demonstrating the impact of formulation adjustments. Additionally, the three‐dimensional response surface plot, generated using Design Expert, provides a visual representation of these interactions, highlighting how formulation components influence solubility, as shown in Figure 3.

**Table 10:** ANOVA quadratic model for solubility response.

Source	Sum of squares	df	Mean square	*F*‐value	*p*‐value	
Model	2638.21	11	239.84	9.21	0.0021	Significant
A‐Carrageenan	2071.62	1	2071.62	79.57	< 0.0001	
B‐Sorbitol	0.0797	1	0.0797	0.0031	0.9572	
C‐Sweet:amaranth	20.06	2	10.03	0.3853	0.6922	
AB	98.51	1	98.51	3.78	0.0876	
AC	1.20	2	0.6014	0.0231	0.9772	
BC	174.43	2	87.21	3.35	0.0877	
*A* ^2^	177.98	1	177.98	6.84	0.0309	
*B* ^2^	7.40	1	7.40	0.2841	0.6085	
Residual	208.28	8	26.03			
Lack of fit	204.14	6	34.02	16.45	0.0584	Not significant
Pure error	4.14	2	2.07			
Cor total	2846.49	19				

Figure 3 illustrates the solubility response data, showing that solubility increases with higher sorbitol concentrations. As a plasticizer, sorbitol reduces intramolecular bonding within polymer chains, weakening the matrix structure and enhancing solubility [30]. Additionally, sorbitol functions as a humectant, binding water and influencing moisture content [31]. The interaction between sorbitol and water occurs through covalent bonding of O and H groups, further promoting solubility [32]. Fu et al. [33] also reported that hydroxyl groups in plasticizers form hydrogen bonds with water, reducing the compactness of the vegetable leather matrix and increasing solubility.

The three‐dimensional surface response curve visually represents this trend, where red regions indicate high solubility, followed by green and blue regions, which reflect a gradual reduction in solubility. However, solubility significantly decreases with increasing carrageenan and sorbitol concentrations [34]. According to Aini et al. [35], higher carrageenan concentrations reduce water content, while increased sorbitol concentrations lead to decreases in water content, total sugar, and titratable acidity. The dissolution rate of plant‐based leather containing carrageenan polymers is influenced by its hydrophilic binding groups. Weaker hydrophilic group binding results in higher solubility, which explains why lower carrageenan concentrations produce more soluble vegetable leather [36]. High solubility indicates lower water resistance and reflects the hydrophilic nature of the vegetable leather [37].

### 3.4. Ash Content

The ash content in vegetable leather is a significant parameter that reflects the inorganic residue remaining after the organic components are burned off. This measure is crucial for understanding the nutritional and compositional attributes of vegetable leather. The ash content can influence the physical and chemical properties of the vegetable leather, affecting its quality and usability [38]. Based on the results of the ANOVA on the tensile strength response data using the Design Expert 13.0.2 program, Table 11 shows that the linear model is the suggested model.

**Table 11:** Model summary statistics of ash content response.

Source	Sequential *p*‐value	Lack of fit *p*‐value	Adjusted *R* ^2^	Predicted *R* ^2^	
Linear	0.0075	0.0662	0.4726	0.1798	Suggested
2FI	0.4283	0.0629	0.4858	−0.7990	
Quadratic	0.5018	0.0564	0.4591	−5.9316	
Cubic	0.0564		0.9586		Aliased

The ANOVA results (Table 12) indicate that the composition of carrageenan, sorbitol, and the sweet leaf–amaranth ratio significantly affects ash content. The ash content primarily originates from the minerals naturally present in the raw materials used, particularly carrageenan, sweet leaves, and amaranth.

**Table 12:** ANOVA linear model for ash content response.

Source	Sum of squares	df	Mean square	*F*‐value	*p*‐value	
Model	42.17	4	10.54	5.26	0.0075	Significant
A‐Carrageenan	41.29	1	41.29	20.58	0.0004	
B‐Sorbitol	1.05	1	1.05	0.5241	0.4802	
C‐Sweet:amaranth	1.27	2	0.6375	0.3178	0.7325	
Residual	30.08	15	2.01			
Lack of fit	29.77	13	2.29	14.53	0.0662	Not significant
Pure error	0.3152	2	0.1576			
Cor total	72.25	19				

Firdaus et al. [39] also reported that higher carrageenan concentrations correspond to increased ash content, which is attributed to the mineral‐rich composition of kappa carrageenan. Consequently, the addition of up to 2.5% carrageenan in this study contributes to the elevated ash content observed in sweet–amaranth vegetable leather compared to previous studies.

Ash content plays a crucial role in influencing the chemical and physical properties of vegetable leather. It contributes to the nutritional value by providing essential minerals and may also affect texture due to its role in matrix formation. However, while minerals can contribute to structural integrity, their direct impact on mechanical properties such as tensile strength and elongation remains uncertain and requires further investigation. In this study, the ash content ranged from 2.18% to 8.87%, reflecting variations in formulation and ingredient composition.

**Figure 4:**
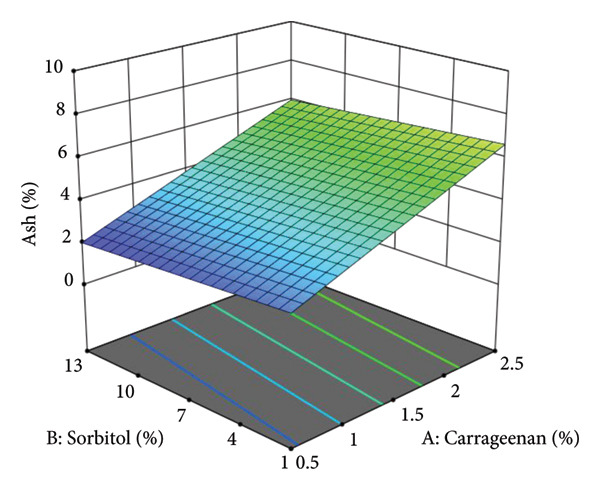
Three‐dimensional surface response curve of ash content.

Figure 4 presents the ash content response data. Besides carrageenan, the graphs indicate that increasing sorbitol concentration leads to a decrease in ash content percentage. The presence of other components, such as sweet leaf, amaranth, and carrageenan, in combination with sorbitol, may also influence the overall composition and ash content of the vegetable leather [40]. This trend is visually represented in the three‐dimensional surface response curve, where blue areas signify lower ash content values, while green to yellow regions indicate higher values.

### 3.5. Moisture Content

**Figure 5:**
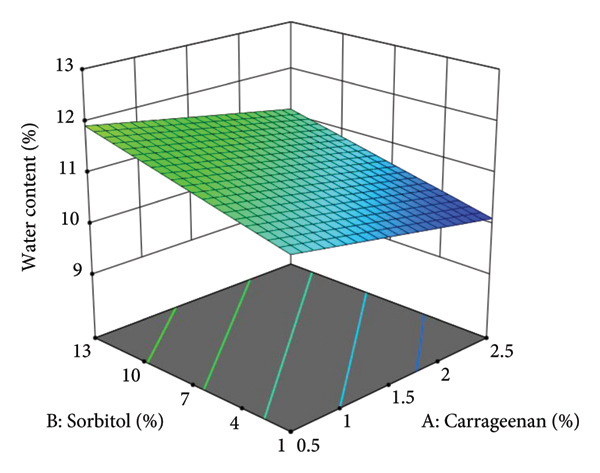
Three‐dimensional surface response curve of moisture content.

Figure 5 presents the three‐dimensional surface response of moisture content. The graph illustrates a decrease in moisture content with increasing carrageenan concentration. According to Aini et al. [35], higher carrageenan concentrations in fruit leather formulations are directly associated with lower moisture content. Carrageenan’s strong gelling properties contribute to this reduction by forming a gel matrix that traps water, thereby decreasing the overall moisture content in the final product [41]. The gelation process in carrageenan occurs through physical cross‐linking, involving noncovalent interactions such as hydrogen bonding and ionic interactions. These interactions are essential for forming a stable three‐dimensional network, effectively immobilizing water molecules [42, 43].

Figure 5 also illustrates a proportional increase in moisture content with higher sorbitol concentrations. This trend is visually represented in the three‐dimensional response curve, where blue regions indicate lower moisture content, while green areas signify higher moisture levels. The moisture content values across all tested formulations range from 10.11% to 12.98%. Sorbitol, widely used in food products for its moisture‐retaining properties, is a polyol that attracts and holds water molecules. This characteristic helps maintain moisture content in food products such as vegetable leather, preventing excessive drying and contributing to overall product stability [44].

### 3.6. Analysis of Optimal Vegetable Leather Formula

Following the analysis of each response parameter, the next stage involved optimization using the Design Expert program to determine the combination of variables that produced the most desirable vegetable leather characteristics. Based on ANOVA results, tensile strength, elongation, solubility, and ash content were identified as significant response variables, making them suitable for inclusion in the predictive model. These parameters showed statistically significant differences (*p* < 0.05), indicating that the independent variables and their interactions strongly influenced the final product properties. Moisture content was excluded from optimization because its variation was not statistically significant at the same confidence level. The optimization criteria applied in the model are summarized in Table 13.

**Table 13:** Optimized vegetable leather response criteria in Design Expert.

Response	Goal	Lower limit	Upper limit	Importance
Tensile strength	Target = 7	3.045	15.552	5
Elongation	Target = 6	2.286	17.797	5
Solubility	Maximize	62.049	98.524	5
Ash content	Minimize	2.181	8.866	3

Using RSM in Design Expert, optimization was conducted to obtain the highest desirability score, which represents the overall balance between mechanical and physicochemical properties. A desirability value close to 1 indicates that the predicted model adequately satisfies all target criteria [12]. From the optimization analysis, 10 potential formulations were generated, with the best formulation achieving a desirability score of 0.864. This optimized formulation consisted of 0.877% carrageenan, 1% sorbitol, and a 25:75 sweet leaf–amaranth ratio. The top 10 predicted formulations are shown in Table 14.

**Table 14:** Solutions formula for vegetable leather in the design expert.

No	Carrageenan	Sorbitol	Sweet:amaranth	Tensile strength	Elongation	Solubility	Ash content	Water content	Desirability
1	**0.877**	**1.000**	**25:75**	**6.078**	**6.000**	**92.769**	**3.109**	**10.928**	**0.864**
2	0.961	1.000	75:25	6.583	6.000	86.933	3.600	10.831	0.838
3	0.960	1.047	75:25	6.575	6.000	86.971	3.594	10.836	0.838
4	0.606	7.518	50:50	5.485	6.000	92.734	2.835	11.131	0.819
5	0.605	7.488	50:50	5.485	6.000	92.734	2.836	11.129	0.819
6	0.606	7.562	50:50	5.484	6.000	92.733	2.834	11.135	0.819
7	0.607	7.622	50:50	5.484	6.000	92.732	2.832	11.140	0.819
8	0.613	8.386	50:50	5.475	6.000	92.725	2.803	11.203	0.819
9	0.620	10.226	50:50	5.424	6.000	92.743	2.718	11.358	0.816
10	2.500	9.935	75:25	11.580	12.444	66.200	6.187	10.997	0.305

Carrageenan concentration played a dominant role in increasing tensile strength and reducing solubility due to the formation of a dense polysaccharide network that improved structural integrity. Higher carrageenan levels slightly decreased elongation, as a stronger gel matrix restricted polymer mobility. Sorbitol, functioning as a plasticizer and humectant, had the opposite effect—it enhanced flexibility and elongation by disrupting hydrogen bonding between carrageenan chains. However, excessive sorbitol tended to weaken the gel structure and reduce tensile strength.

The ratio of sweet leaf to amaranth influenced the pigment retention, ash content, and sensory characteristics of the final product. A higher proportion of amaranth (as in the optimized 25:75 ratio) contributed to deeper color intensity and greater chlorophyll and antioxidant retention. At the same time, sweet leaf improved the cohesiveness and film‐forming ability of the puree due to its natural mucilage content. The combination of moderate carrageenan (0.877%) and low sorbitol (1%) produced a flexible, nonsticky, and uniform sheet with desirable chewability and structural stability. Overall, the optimization model effectively predicted the synergistic effects among carrageenan, sorbitol, and vegetable ratio, confirming their collective contribution to mechanical, functional, and sensory quality.

### 3.7. Chlorophyll and Antioxidant Activity

To further validate the optimized formulation, functional properties related to pigment retention and antioxidant potential were evaluated. The optimized vegetable leather retained a total chlorophyll content of 9.35 ± 0.35 mg·L^−1^, indicating partial preservation of natural pigments despite blanching and extended drying. The relatively high pigment level suggests that mild heat treatment (60°C blanching) effectively minimized chlorophyll degradation. Antioxidant activity measured by the DPPH assay was 76.0 ± 0.02% inhibition, demonstrating strong radical‐scavenging potential. The high antioxidant capacity is attributed to phenolic and flavonoid compounds present in both sweet leaf and amaranth, which are known for their potent antioxidant properties [45–48].

These findings confirm that the optimized formulation successfully retained both pigment compounds and bioactive constituents, supporting its classification as a functional food product. The functional properties obtained here complement the mechanical optimization, showing that the balanced formulation not only improves texture and structure but also preserves nutritional and health‐promoting qualities.

### 3.8. Sensory Evaluation

The optimized formulation achieved favorable sensory characteristics, with mean ± SD scores of 4.4 ± 1.33 (aroma), 4.8 ± 1.07 (taste), 5.1 ± 1.11 (texture), 5.3 ± 1.01 (color), and 5.1 ± 0.90 (overall acceptability) on a seven‐point hedonic scale (1 = very dislike, 7 = very like). These values correspond to neutral to slightly favorable acceptance, with texture and color as the most liked attributes. The soft, pliable texture resulted from the optimal balance between carrageenan and sorbitol concentrations, while the greenish‐brown color reflected partial pigment preservation during drying.

Aroma received the lowest score (4.4), likely due to mild leafy notes inherent to the vegetable base. Incorporating natural flavoring agents or optimizing drying conditions may improve aroma without compromising product safety or stability. Overall, the sensory results align with the mechanical and functional findings, confirming that the optimized vegetable leather offers acceptable consumer quality and promising potential as a functional, shelf‐stable product.

## 4. Conclusion

Optimization of the vegetable leather formulation using RSM produced an optimal composition of 0.877% carrageenan, 1% sorbitol, and a 25:75 sweet leaf–amaranth ratio, achieving a desirability value of 0.864. The optimized product exhibited favorable mechanical and physicochemical characteristics, including adequate tensile strength (6.08 N/mm^2^), elongation (6%), high solubility (92.77%), balanced moisture content (10.93%), and moderate ash content (3.11%).

Functionally, the optimized vegetable leather retained substantial chlorophyll (9.35 ± 0.35 mg·L^−1^) and exhibited potent antioxidant activity (76.0 ± 0.02% inhibition), indicating good preservation of bioactive compounds. Sensory evaluation revealed neutral to slightly favorable acceptance, with texture and color as the most preferred attributes.

Overall, this study demonstrates that sweet leaf and amaranth can serve as effective plant‐based raw materials for producing nutrient‐rich, flexible, and shelf‐stable vegetable leather. The results confirm that combining carrageenan and sorbitol provides a balanced matrix structure with desirable chewability and storage stability. Future research should explore formulation refinement to improve aroma, extended shelf‐life testing, and potential applications in edible films or functional food packaging.

### 4.1. Limitation Statement

Chlorophyll, antioxidant, and sensory analyses were performed only on the optimized formulation, not across the entire design space. Therefore, these functional/sensory results support the optimized formula but cannot be used to infer trends across all treatments. Future work should evaluate these attributes across selected formulation points and examine storage stability.

## Conflicts of Interest

The authors declare no conflicts of interest.

## Funding

No funding was received for this manuscript.

## Data Availability

Data are available upon request from the corresponding authors.
